# The relationship between housing subsidies and supportive housing on neighborhood distress and housing satisfaction: does drug use make a difference?

**DOI:** 10.1186/s13011-016-0064-3

**Published:** 2016-05-27

**Authors:** Julia Dickson-Gomez, Timothy McAuliffe, Chinekwu Obidoa, Katherine Quinn, Margaret Weeks

**Affiliations:** Psychiatry and Behavioral Medicine, Center for AIDS Intervention Research, Medical College of Wisconsin, Milwaukee, WI USA; Global Health, Mercer University, Macon, GA USA; Institute for Community Research, Hartford, CT USA

**Keywords:** Substance use, Supportive housing, Subsidized housing, Neighborhood distress, Homelessness

## Abstract

**Background:**

Since the 1970s, the dominant model for U.S. federal housing policy has shifted from unit-based programs to tenant-based vouchers and certificates. Because housing vouchers allow recipients to move to apartments and neighborhoods of their choice, such programs were designed to improve the ability of poor families to move into neighborhoods with less concentrated poverty. However, little research has examined whether housing voucher recipients live in less distressed neighborhoods than those without housing vouchers. There is much reason to believe that drug users may not be able to access or keep federal housing subsidies due to difficulties drug users, many of whom may have criminal histories and poor credit records, may have in obtaining free market rental housing. In response to these difficulties, permanent supportive housing was designed for those who are chronically homeless with one or more disabling condition, including substance use disorders. Little research has examined whether residents of permanent supportive housing units live in more or less economically distressed neighborhoods compared to low-income renters.

**Methods:**

This paper uses survey data from 337 low-income residents of Hartford, CT and geospatial analysis to determine whether low-income residents who receive housing subsidies and supportive housing live in neighborhoods with less concentrated poverty than those who do not. We also examine the relationships between receiving housing subsidies or supportive housing and housing satisfaction. Finally, we look at the moderating effects of drug use and race on level of neighborhood distress and housing satisfaction.

**Results:**

Results show that low-income residents who receive housing subsidies or supportive housing were not more or less likely to live in neighborhoods with high levels of distress, although Black residents with housing subsidies lived in more distressed neighborhoods. Regarding housing satisfaction, those with housing subsidies perceived significantly more choice in where they were living while those in supportive housing perceived *less* choice. In addition, those with rental subsidies or supportive housing reported living closer to needed services, unless they also reported heavy drug use.

**Conclusions:**

Housing subsidies and supportive housing have little impact on the level of neighborhood distress in which recipients live, but some effects on housing satisfaction.

## Background

It is widely agreed that there is a shortage of affordable housing in the United States. Approximately 17 million households (approximately 1 in 7) are severely rent burdened (defined as spending over 50 % of their income on housing) [10] and approximately 1 % of the nation’s population experience homelessness in a year [25]. At the same time, many researchers have argued that living in racially segregated, impoverished neighborhoods with high levels of crime and social disorder contribute to health and economic disparities, substance abuse, violence and poor school outcomes [1, 5, 11, 21, 32]. Since the mid-1970s, the dominant model for U.S. federal housing policy has shifted from unit-based programs to tenant-based vouchers and certificates. This shift occurred in response to the negative views of public housing, the deteriorated condition of many public housing buildings, and as an effort to de-concentrate poverty in inner-city neighborhoods [1, 18, 20, 31]. Since the 1990s, many public housing projects have been demolished and replaced by mixed-income housing. Mixed income housing is a deliberate effort to construct housing for both low and moderate income groups [2]. They are often the result of private non-profit and public partnerships and can receive funding from federal, state or local sources.

Because housing vouchers allow recipients to move to apartments and neighborhoods of their choice, such programs were designed to improve the ability of poor families to move into neighborhoods with less concentrated poverty. At the same time, mixed income housing projects built to replace public housing projects were intended to increase the income mix of impoverished neighborhoods. Both these actions were intended to provide poor, inner-city residents more “bridging social capital” based on the idea that middle class families may give low-income residents more contact to gainful employment and educational opportunities [13].

Surprisingly little research has examined the question of where those with housing vouchers are able to live in real life circumstances. While some studies have found that housing voucher holders are less likely to live in distressed neighborhoods than public housing residents [1, 19, 28], other research has found that voucher holders are more likely to reside in distressed neighborhoods than unsubsidized renter households [19, 20], particularly among African American voucher holders [20].

There are several potential explanations for the continued concentration of voucher holders in distressed neighborhoods. One important reason may include federal, state and local policies that exclude drug users or their family members from receiving or maintaining Housing Choice vouchers. The federal “One Strike and You’re Out” law (P.L. 104–120, Sec.9), passed in 1996, allows federal housing authorities to consider drug and alcohol abuse and convictions by people and their family members when making decisions to evict them from or deny access to federally subsidized housing. Importantly, many state Public Housing Authorities, including Connecticut, ignore this law. However, housing choice voucher holders must find and obtain free-market rental housing, many of which require criminal background checks. While African American men comprise less than three percent of Connecticut’s population, they account for 47 % of the state’s inmates in prisons, jails and halfway houses [22]. Thus, the disproportionate incarceration of poor, inner-city, ethnic minority residents, particularly African American men, further limits the extent to which they may utilize rental subsidies to obtain free market rental housing.

In addition, there may be an inadequate supply of low-cost rental housing in private control. Low-cost rental housing may be concentrated in distressed neighborhoods, while some more affluent communities, particularly suburban communities, may have housing markets dominated by single family homes [20]. Researchers who have studied low-income voucher holders’ efforts to rent have shown that landlords in more affluent areas have little incentive to accept housing choice vouchers. Many have negative perceptions of voucher holders, including that voucher holders have substance use problems, and may feel that the paperwork and inspection process of voucher programs is too cumbersome [7, 8, 20, 35]. The demand created by housing vouchers may be insufficient to substantially increase housing stock [1, 20]. Housing vouchers alone do not eliminate racial segregation and discriminatory renting [18–20].

In more recent years, U.S. housing policy has concentrated on ending chronic homelessness by providing affordable, service-enriched rental housing for homeless and at-risk people, many of whom suffer from mental health and substance abuse problems [3]. Like rental housing vouchers, however, these programs are woefully underfunded. The majority of funding for the housing units comes from HUD or state sources, while services are often paid for by Ryan White HIV/AIDS program for people living with HIV or the Substance Abuse and Mental Health Services Association (SAMSHA). Different states have more quickly adopted Supportive Housing models. However, supportive housing is an umbrella term that covers programs that may differ in important characteristics such as whether apartments are located in a single building or in fair market rental units scattered throughout cities. Other programs may insist on abstinence from drugs and alcohol, making it difficult for those with problematic substance use to maintain their housing [29]. While some research has been conducted to examine the effectiveness of supportive housing in increasing housing stability among the chronically homeless [12, 14, 23, 33], little research to date has examined characteristics of neighborhoods in which supportive housing residents live. However, one study suggests that supportive housing for the psychologically disabled is more likely to be located in more distressed neighborhoods than supportive housing for the developmentally disabled [34].

The level of crime and disorder in a neighborhood is not the only factor that determines how satisfied people are with their overall housing or how well they do psychologically or socially. In addition to being more satisfied when they felt that they were safe in their neighborhoods and homes, studies have shown that housing satisfaction also depends on degree of choice that individuals felt they had in choosing where they live, and whether they are close to services and people such as family [27]. Thus, the degree to which moving to safer neighborhoods may improve individuals’ housing satisfaction may be mitigated to the degree to which they feel that they were able to choose their current housing and neighborhood and their proximity to important social networks and resources.

This paper draws from data that explored the effect of substance use on drug using and non-drug using low-income residents’ access to housing, and how housing status affected their HIV risk. In this paper, we examine whether low-income residents who receive housing choice vouchers or supportive housing live in less distressed neighborhoods than residents who receive no subsidies or supportive housing. The paper will address the following questions.

1. Are neighborhoods where low income people with housing choice vouchers live in greater proportion to low income residents without vouchers less distressed neighborhoods?
2. What are the moderating effects of neighborhood race/ethnicity, gender, criminal history, eviction and recent drug use rates on the relationship between the percentage of people having rental vouchers or supportive housing who live in the neighborhood and neighborhood distress?

Because of racial discrimination in housing markets, and the increased difficulty that substance users, those with criminal histories or evictions may have in competing for the most desirable free market rental housing, we expect that voucher holders with recent substance use, criminal histories or evictions will live in neighborhoods with more economic distress than those without these characteristics.

3. Do people in subsidized or supportive housing report more satisfaction with their housing in terms of housing choice, safety, proximity to important others, and privacy?

## Methods

### Study site

This study was conducted in Hartford, Connecticut a small city with high rates of poverty and a majority ethnic minority population. Connecticut was an early adopter of Supportive Housing Programs, but far fell short of their 10 year plan to provide 10,000 units by 2010 due to the housing and economic crisis in 2008 (Partnership for Strong Communities, 2010).

### Study population

Study participants were 337 low-income residents of Hartford recruited through a targeted sampling plan [30] between October 2008 and August 2010. We sampled both current drug users, defined as using heroin, cocaine or crack in the last 30 days, or non-drug users, defined as having used none of these substances in the last year. This was in order to compare the effect of drug use on access to housing subsidies, supportive housing or free-market rental housing. Formative research was conducted that reviewed 2000 census data to identify low-income block groups, data from town property assessors, the town planning department and other sources to identify areas in Hartford that have experienced significant change in housing stock characteristics since the 2000 census, for example demolition of large housing projects that would significantly reduce the number of residents in the census block. Windshield surveys—driving or walking in neighborhoods to note conditions of housing, streets, public places and the number of abandoned buildings—were conducted in high poverty block groups to further identify changes in housing stock and identify recruitment locations, and key informant interviews. The windshield surveys revealed that the demolition of low-income housing projects and replacement with commercial properties had resulted in almost no residents in two census tracks. These census tracks were subsequently eliminated from the targeted sampling plan. Otherwise, census data were used to develop the sampling plan to target recruitment in appropriate geographic areas and recruit a sample representative of low-income residents. The targeted sampling plan indicated the expected number of low-income residents by gender and ethnicity in each census block group in Hartford that should be reflected proportionately in our study sample. Field staff approached potential participants in venues in different census blocks in order to reach a sample that represented the geographic and racial/ethnic diversity in the city. Venues included supermarkets, bus stops, on the street and homeless shelters and soup kitchens for those census blocks which had them.

Potential participants were screened for eligibility after obtaining informed consent due to the sensitivity of some of the questions to determine eligibility. We used the Department of Housing and Urban Development’s definition of low-income, based on 50 % or less of the median income for a family of four in the Hartford metropolitan area, adjusted according to household size. During screening, potential participants were asked to report their town/city of residence, their annual household income, their household size, and a drug matrix asking about lifetime use of a number of different drugs including heroin and cocaine, and, in cases when respondents indicate that they have consumed a particular drug in their lifetimes, the last time they used that drug. Those who do not meet HUD’s definition as low-income were ineligible for the study. In addition, we used a quota system to ensure equal numbers of drug users and non-drug users in the sample. However, in order to over-sample participants who resided in supportive housing programs, all supportive housing residents (by definition low-income) were eligible to participate in the project regardless of drug use. As a result, many had used drugs in the past year but not the past 30 days. Thus, in analyses, we used the variable recent hard drug use (cocaine, heroin or crack in the last 30 days). All participants gave their signed informed consent. Study protocol was approved by the Institutional Review Boards of the Medical College of Wisconsin, Milwaukee, WI (Protocol number 0008184) and the Institute for Community Research, Hartford, CT.

### Measures

#### Survey

As part of the survey, participants were asked to provide the addresses of the locations where they currently reside as well as addresses of other residences in which they lived in the past 6 months. This paper reports on current residence only. Participants were also asked how they would best describe the location where they currently reside: 1) homeless on the street; homeless in a shelter; 2) “doubling up” or temporarily living in a sex partner’s apartment; 3) doubling up in the apartment of a friend or acquaintance; 4) doubling up in a family member’s apartment; 5) living in own apartment with no rental subsidy; 6) living in own apartment with a rental subsidy (such as Section 8, Housing Choice Vouchers); or 7) living in supportive housing, (housing that is paid for by a rental subsidy and includes supportive services). In this paper we identified those who were in supportive housing or housing paid for by a subsidy separately for comparison with those in who were doubled up with family, sex partners or acquaintances, or living in their own apartments without a subsidy, or homeless.

Personal/demographic characteristics measured included race/ethnicity, gender, history of incarceration, self-reported mental illness, HIV status, income, level of education, marital status and use of crack, heroin or cocaine in the last 30 days.

Housing status and neighborhood satisfaction measures were drawn from the SAMHSA Housing Satisfaction scale, which has been used in previous tests with formerly homeless, mentally ill participants [26]. Response categories were on a four-point scale, ranging from strongly disagree to strongly agree. Housing satisfaction was measured using the item, “I am very satisfied with the place I live.” Access to social services was measured using the item, “The place I live is close to the social services I need.” The survey also assessed living close to important people (“I live close to people who are important to me”) and isolation (“I feel isolated in the place I live”). Finally, to assess housing choice we asked participants to what degree they were able to choose the place where they live. For our analyses, responses were dichotomized as disagree or agree.

#### Secondary data sources

We retrieved socio-demographic, economic and housing data from the 2006 to 2010 American Community Survey 5-year estimates. http://factfinder2.census.gov/faces/nav/jsf/pages/index.xhtml. We used census tract boundaries to define neighborhoods in the city. Data used in this analysis include 8 items measuring economic distress and housing characteristics. Economic distress included: 1) percentage of people unemployed, 16 years and older, civilian and in the labor force; 2) percentage of people with less than a 9^th^ grade education; 3) percentage of people earning less than $10,000 per year; 4) percentage of households receiving cash benefits; 5) percentage of people receiving food stamps; 6) percentage of people receiving supplemental security income; 7) percentage of female heads of household, with no husband present and with related children under 18 years old; and 8) the percentage of vacant housing units.

Spatial data constituted the Hartford census tracts shape file and the Hartford town boundary shape file which were downloaded from the virtual geographic database of the Map and Geographic Information Center at the University of Connecticut. No special permission was required to access and use this data (http://magic.lib.uconn.edu/connecticut_data.html#featured). The percentages were summed to produce an overall distress score with a possible maximum score of 800. Higher scores indicated more distress, e.g., percentage of female-headed households with children under the age of 18.

### Data analysis

Participants’ census tract and distress value were determined through the spatial join procedure in ArcGIS that joins the point file (location of participants in the study) with the polygon shape file (census tract). The join produces a layer and table that indicate which census tract each point (individual) falls in. Other census tract level attributes are also linked to the participants through this procedure.

We used multiple linear regressions to investigate the association between housing and participant attributes and living in a neighborhood with a greater level of distress. Our analyses are conducted at the census tract level. We aggregated the survey data for all respondents living within each census tract. We calculated the means for numeric data (e.g., mean age) and the percentages for categorical data (e.g., proportion Black). We first performed backwards stepwise regression including the aggregate demographic variables for age, Black race, female gender, less than high school education, mental health or HIV-positive diagnosis, any recent hard drug use, any criminal conviction, receiving supportive housing and receiving subsidized housing to predict living in a neighborhood with a greater level of distress. We also included interactions between Black race, recent hard drug use and criminal conviction with receiving supportive housing and with receiving subsidized housing. The final backwards stepwise regression model included the two variables mental health diagnosis and the interaction of Black race and receiving subsidized housing. We then performed a standard multiple linear regression using only the variables that remained significant in the stepwise regression and the main factors involved in significant interactions to examine neighborhood level of distress.

Finally, we used multiple logistic regression to examine the relationship between housing status and housing and neighborhood satisfaction. Housing status was categorized as the following: living in own home/apartment without rental assistance (reference category); doubled up (living with a family member, a friend, or a sex partner); living in own home/apartment with a rental subsidy; or living in a supportive housing program. We dichotomized participant satisfaction (e.g., satisfied with housing, living close to people important to me, my neighborhood is a safe place to live) as agree or disagree. These analyses are performed using individual-level data. We performed multiple logistic regressions to compare the reference housing group to the other housing status groups, separately. Covariates included in the logistic regression models were Black race, Female gender, less than high school education level, mental health diagnosis, HIV-positive status, and hard drug use in the past 30 days. Hard drug use over the previous 30 days was assessed by the number of times crack, cocaine, or heroin were used by the participant in the past 30 days. We also tested for significant interactions of race with housing status and of hard drug use and housing status in the models.

## Results

### Descriptive statistics

A total of 337 Hartford residents constituted the sample for the spatial analysis. Sixty-five (19 %) of the participants indicated that they lived in subsidized housing units and 51 (15 %) in supportive housing units. Most participants were African American or Latino and had a criminal history. Neighborhood distress is relatively high in the city. The distress scores ranged from 54.9 to 266.7; Mean 180.8; Standard deviation 56.4. In neighborhoods scoring the mean on neighborhood distress, 20 % or more of residents would be categorized in each of the distress indicators, i.e., 20 % making less than $10,000 a year, 20 % with less than a 9^th^ grade education, etc. Participant demographics and receipt of subsidized or supportive housing are presented in Tables 1 and 2.

**Table 1:** Sample^a^ characteristics

Characteristic	% (*n*) unless otherwise specified
Age (years), mean (SD)	45 (8)
Gender^b^	
Male	66 % (222)
Female	34 % (114)
Race/Ethnicity^b^	
African American	34 % (116)
White-not Hispanic	16 % (55)
Puerto Rican or other Latino	48 % (162)
Criminal conviction	64 % (216)
Receiving Supportive Housing	15 % (51)
Receiving Housing Voucher	19 % (65)
Doubled-up with family, friend or sex partner	28 % (95)
In own apartment with no rent subsidy	12 % (42)
Less than HS education	48 % (161)
Mental health diagnosis	50 % (168)
Told by a doctor you have HIV	27 % (90)
Any drug use in past 30 days	60 % (204)

^a^Total *N* = 337

^b^Excludes 1 transgender and 4 or mixed or other race

**Table 2:** Multiple linear regression of neighborhood level of distress

Variable	Coefficient (SE)	β	t	*p*-value
Black (%)	.140 (.321)	.072	0.44	.66
Mental health diagnosis (%)	−.791 (.408)	−.329	−1.94	.061
Receipt of subsidized housing (%)	−.230 (.508)	−.083	−0.45	.65
Black*Receipt of subsidized housing (%)	2.462 (.989)	.434	2.49	.018

Characteristics of participants within each census tract (e.g., percent Black)

Census-tract level analysis: Number of census tracta = 37; Fitted model *F* = 3.16; df = 4, 32; *p* = .027; R-square = .28

### Spatial analysis

#### Distribution of neighborhood distress

Descriptive cartographic analysis of the distribution of neighborhood distress indicated a marked pattern. High values of distress were concentrated in the north and south central parts of the city. Spatial autocorrelation of the neighborhood distress index revealed a strong significant pattern (Z = 3.49; *p* = <.0001); Moran’s Index = 0.233822.

#### Association of subsidized and supportive housing and neighborhood distress

Backward stepwise regression indicates that in general neighborhood racial distribution or percentage receiving supportive or subsidized housing were not significantly related to neighborhood level of distress. In general, neighborhoods with higher percentages of residents who received supportive or subsidized housing were neither more nor less distressed neighborhoods. However, there was a significant interaction between neighborhood racial distribution and percentage of residents who received subsidized housing, with neighborhood with higher percentages of African Americans who received subsidized housing more likely to be highly distressed neighborhoods. Those neighborhoods in which a higher percentage of survey respondents having a mental health diagnosis lived were also more likely to be less distressed neighborhoods. Neighborhood gender, education, HIV status, drug use, and criminal conviction rates were not associated with neighborhood distress.

#### Logistic regression analysis of housing status and neighborhood satisfaction

Given that African Americans who receive subsidized housing were more likely to live in higher distressed neighborhoods, we wanted to further examine individual’s satisfaction with their housing and neighborhood. Results from the logistic regression analyses adjusted for race (Black), gender, education (less than high school), mental health diagnosis, HIV status, and recent hard drug use compared supportive, subsidized or doubled-up housing to living in their own apartment/home without rental assistance indicate that Black individuals living in supportive housing were significantly more likely to report living in a safe neighborhood (adjusted odds ratio (AOR) 12.2, 95 % CI: 1.32–112.3) as compared to Blacks living in their own apartments without a subsidy. Although in general individuals living in supportive housing reported having less choice in where they live (AOR 0.20, 95 % CI 0.40–0.95), they were more likely to report living close to needed social services (AOR 4.58, 95 % CI: 1.32–45.9). Individuals who received rental subsidies were significantly more likely to live close to needed social services (AOR 9.28, 95 % CI: 1.83–47.0). However, there was a significant interaction between receiving subsidies and recent drug use with individuals who received rental subsidies and reporting more frequent hard drug use in the past 30 days were significantly less likely to live close to needed social services (AOR 0.09, 95 % CI: 0.01–0.71), than individuals living alone without subsidies. Individuals who doubled up with a family member, a friend, or a sex partner tended to be less likely to report living in a safe neighborhood (AOR 0.32, 95 % CI: 0.08–1.31), and those who also reported having used higher levels of hard drugs in the past 30 days were significantly less likely to live close to needed social services (AOR 0.20, 95 % CI: 0.04–1.20). However, there was an interaction between race and housing status with African Americans who lived doubled up reporteing more choice in where they live (AOR 8.28, 95 % CI 1.26–54.7), greater satisfaction with their housing (AOR 25.9, 95 % CI: 3.55–188.6), and living in a safe neighborhood (AOR 10.7, 95 % CI: 1.84–62.0) as compared to African Americans living in their own apartments without a subsidy (see Table 3).

**Table 3:** Results of Logistic Regressions Predicting Housing and Neighborhood Satisfaction Comparing Supportive, Subsidized and Doubled-up Housing with Non-Subsidized Rental Housing

Satisfaction	Housing status*	Adjusted Odds Ratio	*P*	Interactions with housing	
		AOR (95 % CI)		Factor	AOR (95 % CI)
Had a choice in where to live					
	Supportive housing	0.20 (0.40–0.95)	0.043		
	Rental subsidy	1.26 (0.25–6.37)	0.782		
	Doubled up	0.62 (0.13–3.03)	0.552	Black	8.28 (1.26–54.7)
Live close to social services					
	Supportive housing	4.58 (1.32–45.9)	0.058		
	Rental subsidy	9.28 (1.83–47.0)	0.007	Drug use	0.09 (0.01–0.71)
	Doubled up	2.75 (0.64–11.87)	0.174	Drug use	0.20 (0.04–1.20)
Satisfied with housing					
	Supportive housing	1.69 (0.28–10.25)	0.569		
	Rental subsidy	1.57 (0.32–7.79)	0.583		
	Doubled up	0.32 (0.07–1.50)	0.150	Black	25.9 (3.55–188.6)
Live close to important people					
	Supportive housing	0.37 (0.08–1.62)	0.188		
	Rental subsidy	0.51 (0.13–2.05)	0.341		
	Doubled up	0.66 (0.15–2.93)	0.586		
Neighborhood is a safe place to live					
	Supportive housing	1.81 (0.34–9.50)	0.484	Black	12.2 (1.32–112.3)
	Rental subsidy	0.90 (0.23–3.54)	0.876		
	Doubled up	0.32 (0.08–1.31)	0.113	Black	10.7 (1.84–62.0)

*Housing groups Supportive housing (*n* = 49), Rental subsidy (*n* = 65), and Doubled up with a friend, family, or sex partner (*n* = 95) are compared with the referent group In own apartment without a rental subsidy (*n* = 42). Satisfaction is categorized as agree or disagree. Covariates are Black race, female gender, less than high school education, mental health diagnosis, HIV-positive, any illegal drug use in past 30 days, and interactions housing status with race and housing status with drug use

## Discussion

Hartford has neighborhoods with concentrated levels of distress. However, neighborhoods with residents with more housing subsidies or supportive housing were neither more nor less likely to be neighborhoods with a greater level of distress than those with fewer subsidies. This may indicate that receiving supportive housing or housing subsidies is not sufficient to help low-income residents move out of impoverished neighborhoods, although results should be interpreted with caution. This result has been found in some research that found that voucher holders were no more likely to live in neighborhoods with less distress than other renter households [19, 20], but contradicts other research that found that voucher holders were less likely to live in distressed neighborhoods than low-income residents without vouchers [1, 28], This may be due to differences in the cities studies. Hartford is a small city with a high level of economic distress throughout its neighborhoods and, as a whole, may not be seen as a desirable place to live. In a city such as Hartford, therefore, having a housing subsidy may have little overall impact on whether or not residents are able to move out of distressed neighborhoods. The differences in our results may also be due to differences in our study design compared to previous studies. In our study, we sampled members of low-income residents and looked at whether neighborhoods with a greater or lesser proportion of low-income residents of supportive or subsidized voucher holders are more or less distressed. In order to answer the question of whether voucher holders are more or less likely to live in neighborhoods with higher or lower levels of distress, we would need to sample from the population of voucher holders with a comparison sample of non-voucher holders.

This study did not find that more distressed neighborhoods were more likely to have a greater proportion of participants with recent substance use, criminal histories or evictions. In previous analysis of these data, conducted at the individual not neighborhood level, we found that none of these factors predicted access to housing subsidies or supportive housing [9]. In part, this may be because Connecticut public housing authority has elected not to consider criminal drug convictions as disqualifying conditions for receiving or maintaining Housing Choice vouchers. Access to housing subsidies and supportive housing may have eliminated any effects that drug use, prior evictions or criminal records may have had on the quality of neighborhood in which drug users resided. Perhaps equally important, the vast majority of our participants, both recent drug users who had consumed in the past 30 days and those who had not consumed in over a year, had criminal convictions and our “non-drug using” participants often had histories of using illicit substances albeit over a year ago. Therefore, we may not have had the power to detect differences in proportion of residents with criminal convictions or illicit drug use.

Supporting results seen in the study by Pendall [20], we found a significant interactive between neighborhood racial distribution and percentage of residents who received housing subsidies on neighborhood distress. In our study, neighborhoods with greater proportions of African Americans who receive housing subsidies were more likely to be neighborhoods with higher degrees of distress. African Americans who receive housing subsidies may experience greater discrimination due to the double stigma of being a racial minority and also a housing subsidy recipient. Qualitative studies have shown that landlords tend to suspect that housing voucher holders will have problems with drug use, mental illness and generally be undesirable tenants [17].

Regarding neighborhood/housing satisfaction, those in supportive housing were about a fifth as likely to perceive they had a choice in where they lived while those who received a rental subsidy were 1.3 times more likely to feel they had a choice of where to live Unlike other studies [26], participants in our study who resided in supportive housing felt less choice in where they were allowed to live. Supportive housing programs in Connecticut consist of both fixed and scattered site housing. It is not surprising that those residing in fixed site supportive housing programs (approximately half of those in supportive housing in our sample) would perceive little choice in their housing or neighborhood, as all housing units are often located within a single building. However, those in scattered site housing may also have less choice in housing than intended by supportive housing providers. In scattered site supportive housing, supportive housing staff have reported developing relationships between their programs and particular landlords. In in-depth interviews, they describe gaining the trust and support of landlords to house residents with substance use, HIV or mental illness by assuring them that their program will intervene with residents when problems occur [6–8]. While these kinds of personal relationships may be necessary in order to convince reluctant landlords to accept supportive housing residents, in the process supportive housing providers may be steering their clients to “friendly” landlords, thus limiting their choice. However, those who received subsidized housing felt more choice in their housing. Thus, whether or not they are able to live in more or less distressed, they felt that the subsidy gave them more choice of where to live compared to low-income residents who didn’t receive a subsidy.

Those residing in supportive housing programs were 4.5 times and those who received subsidized housing were 9.3 times as likely to report living near to social services. However, those who received subsidized or supportive housing who also reported using more drugs in the last 30 days were less likely to report living next to social services that they needed, unlike previous studies [16]. This may be because heavy drug users were less able to access services that they needed, regardless of where they lived. Also unlike other studies, those who received supportive housing or housing subsidies were not more likely to report being satisfied with their housing [27]. However, African Americans who were doubled up were 25 times more likely to be satisfied with their housing than others. The African American community in Hartford report large extended family, including “fictive kin” or close friends who take on kinship status that help low-income African Americans survive in precarious economic situations [24]. Thus, those who doubled up may have been living with actual or fictive kin who provided social and instrumental support. It is also possible that by pooling resources and having more adults to contribute to rent, participants who reported doubling up lived in better quality housing. Finally, there were no significant effects of housing status on how safe participants perceived their neighborhoods to be. However those who were African American and living in supportive housing and those who were Black and living doubled up reported that they lived in safer neighborhoods. Similar, to the explanation above, African Americans who doubled up may have been able to pool their resources to live in housing in safer neighborhoods. It is not clear why African Americans who lived in supportive housing perceived their housing to be in safer neighborhoods since there was no interactive effect of race and receiving supportive housing on actual neighborhood distress. Perceptions of safety may have more to do with characteristics of the housing, having limited access to the building, or the amount of crime in the neighborhood which was not reflected in our distress index.

### Limitations

This study has some limitations. The cross-sectional design of the study found an association between receiving housing subsidies and being African American and living in more distressed neighborhoods, housing status and measures of housing satisfaction, and interactions between housing status, drug use and race and housing satisfaction measures but could not determine causation. Further limiting interpretation of our results, we did not ask participants how long they had received their rental subsidy or supportive housing. It may be the case that those who have received rental assistance for longer periods of time are able to leverage past success as tenants to move into better neighborhoods. Conversely, rental subsidy holders may, over time, move back to higher distress neighborhoods closer to where they originally lived and where family members still reside [21]. Other limitations include the small sample size and the fact that the study was conducted in a small city.

## Conclusions

This study is the first to examine where low-income residents who receive rental subsidies or supportive housing live compared to those who do not receive housing assistance in a small New England city. Our participants received no aid beyond that which is customarily provided to voucher holders, and had no restrictions on their choice of neighborhood, unlike other projects with quasi-experimental designs [4, 15, 21]. Thus, our study more closely matches the real life circumstances in which low-income people receive and use housing subsidies or supportive housing. The current need for affordable housing—whether through traditional public housing projects, housing choice vouchers or supportive housing programs—far outmatches its availability. It is not clear whether increasing the number of housing choice vouchers would saturate the market, thereby reducing the “choice” of neighborhoods provided by rental vouchers. This may explain some of the mixed results of rental subsidies on moving out of areas of high distress. Different cities vary in their rental markets and housing pressures, which also may change over time. In tight housing markets with few available units in desirable neighborhoods and high rents, landlords may be unlikely to accept housing choice vouchers, choosing instead to rent to tenants with high paying jobs. Different cities also have different histories of ethnic/racial segregation. Future research of this sort is needed in a variety of cities of differing sizes and in different geographic regions of the United States. Such research could help identify large structural factors such as segregation, income disparities, and the housing market, that may determine the relative mobility or segregation of rental subsidy holders.
